# Excipient Interactions in Glucagon Dry Powder Inhaler Formulation for Pulmonary Delivery

**DOI:** 10.3390/pharmaceutics11050207

**Published:** 2019-05-01

**Authors:** Md Abdur Rashid, Amged Awad Elgied, Yahya Alhamhoom, Enoch Chan, Llew Rintoul, Ayman Allahham, Nazrul Islam

**Affiliations:** 1 Department of Pharmaceutics, School of Pharmacy, King Khalid University, Abha, Aseer 62529, Saudi Arabia; amged911@gmail.com (A.A.E.); ysalhamhoom@kku.edu.sa (Y.A.); 2 Pharmacy Discipline, School of Clinical Sciences, Faculty of Health, Queensland University of Technology, Brisbane, QLD 4000d, Australia; enoch.chan@qut.edu.au; 3 School of Chemistry, Physics and Mechanical Engineering, Science and Engineering Faculty, Queensland University of Technology, Brisbane, QLD 4000d, Australia; l.rintoul@qut.edu.au; 4 Pharmacy Program, School of Health and Biomedical Sciences, College of Science, Engineering & Health, RMIT University, Bundoora, VIC 308, Australia; ayman.allahham@rmit.edu.au; 5 Institute of Health and Biomedical Innovation, Queensland University of Technology, Brisbane, QLD 4000d, Australia

**Keywords:** glucagon, dry powder inhaler formulation, pulmonary drug delivery, excipients interactions, fine particle fractions, FTIR, DSC

## Abstract

Purpose: This study describes the development and characterization of glucagon dry powder inhaler (DPI) formulation for pulmonary delivery. Lactose monohydrate, as a carrier, and L-leucine and magnesium stearate (MgSt) were used as dispersibility enhancers for this formulation. Methods: Using Fourier-transform infrared (FTIR) spectroscopy, Differential Scanning Calorimetry (DSC), and Raman confocal microscopy, the interactions between glucagon and all excipients were characterized. The fine particle fractions (FPFs) of glucagon in different formulations were determined by a twin stage impinger (TSI) using a 2.5% glucagon mixture, and the glucagon concentration was measured by a validated LC-MS/MS method. Results: The FPF of the glucagon was 6.4%, which increased six-fold from the formulations with excipients. The highest FPF (36%) was observed for the formulation containing MgSt and large carrier lactose. The FTIR, Raman, and DSC data showed remarkable physical interactions of glucagon with leucine and a minor interaction with lactose; however, there were no interactions with MgSt alone or mixed with lactose. Conclusion: Due to the interaction between L-leucine and glucagon, leucine was not a suitable excipient for glucagon formulation. In contrast, the use of lactose and MgSt could be considered to prepare an efficient DPI formulation for the pulmonary delivery of glucagon.

## 1. Introduction

Glucagon, a biologically active peptide, is known to be effective for the treatment of insulin-induced hypoglycemia and may be used to maintain the normal circulating glucose level in patients with pancreatectomy. Currently, treatment for severe hypoglycemia is limited to intramuscular injection of a glucagon formulation, which is commercially available as an emergency kit consisting of lyophilized glucagon powder that must be mixed with diluents immediately prior to injection. This preparation and administration procedure can be inconvenient, intimidating, and is more susceptible to potential dosing errors, especially when administered by people who are not trained in reconstitution and injection techniques in emergency situations. Recently, nasal delivery of glucagon powder has been investigated for treating diabetes-related severe hypoglycemia [1,2]. It consisted of a dry powder spray formulation of synthetic glucagon in a single-use device. These studies demonstrated that the nasal formulation was as efficacious and safe as an intramuscular glucagon product for the treatment of insulin-induced hypoglycemia in adults [2]. The authors suggested that nasal glucagon can be used as a rescue treatment for severe hypoglycemia but noted unavoidable side effects that included itching of the eyes, throat, and nasal lining. Therefore, a safe, efficacious, alternative route of administration for the delivery of a stable formulation of glucagon is required. 

Pulmonary delivery of drug formulations using a dry powder inhaler (DPI) is an alternative to invasive injections and has been found to be the most promising non-invasive route for drug administration [3,4,5]. In addition to avoiding first-pass metabolism, administration via inhalation has several apparent benefits that are attributable to the large surface area within the lungs (100 m^2^), the ultra-thin alveolar epithelium (0.1–0.5 μm), and the low metabolic enzyme activity. Recently, systemic delivery of proteins and peptides by inhalation has attracted significant attention due to the promising advantages of this route for drug delivery. Endo et al [6] studied an erythritol-based DPI formulation of glucagon that had high flowability and dispersibility as a powder mixture. Intratracheal administration of the glucagon dry powder to anesthetized rats improved transpulmonary absorption of glucagon resulting in increased blood glucagon and sugar levels. These results suggest the usefulness of an erythritol-based powder form of glucagon for systemic administration. A further study using a DPI formulation of glucagon combined with citric acid showed a three-fold increase in the hyperglycaemic effect in rats when compared to a glucagon DPI without citric acid [7]. An additional rat model using glucagon loaded PLGA nanospheres produced a fine particle fraction (FPF) of 20.5% with sustained release, leading to a prolonged hyperglycaemic effect [8]. 

Lactose is a well-established inert excipient and has been used as a carrier in DPI formulations in order to improve the flow of the particles, resulting in increased delivery efficiency of formulations [9,10]. Excipients such as MgSt [11,12,13,14,15] and leucine [16,17,18] are used in formulations as dispersibility enhancers. Almost all of these excipients are believed to be compatible with most drugs; however, drug excipient interactions in most formulations have not been fully investigated. 

Raman microscopy, a non-destructive analytical technique, has previously been used to visualize the component distribution of powder mixtures [19]. Very recently, Afrina et al. demonstrated the application of Raman spectroscopy to investigate the interactions and distribution of ibuprofen crystallized with other additives [20]. Fourier-transform infrared spectroscopy (FTIR) was used to study the interactions of powders (drug and excipients) in interactive mixtures [21,22]. Using FTIR, Fang et al [21] investigated the stability of freeze-dried glucagon with certain excipients in order to develop a stable formulation of glucagon. Successful application of FTIR in determining the denaturation of secondary protein structure [23] and the stability of freeze-dried PEGylated glucagon [22] have been demonstrated. Thus, the purpose of this study was to investigate the stability of glucagon in interactive mixtures of glucagon, lactose, leucine, and magnesium stearate (MgSt) with the aim of developing an efficient DPI formulation for pulmonary glucagon delivery. While the use of inhalable micronized lactose as a carrier, and the use of leucine and MgSt as dispersibility enhancers in DPI formulations, has been established, no FTIR studies have investigated the possible interactions between glucagon and these excipients. Therefore, the objective of this study was to investigate the interactions between glucagon hydrochloride, and excipients (lactose, leucine, and MgSt) by determining their structural integrity and the stability of glucagon in the powder mixtures for the optimization of a DPI formulation. 

## 2. Materials and Methods

### 2.1. Materials 

Glucagon hydrochloride and L-leucine were purchased from Sapphire Bioscience (Redfern, NSW, Australia) and RMIT University (Melbourne, Australia), respectively. Inhalation grade lactose monohydrate (Inhalac 120) was donated by Meggle GmbH (Wasserburg, Germany).

### 2.2. Methods

#### 2.2.1. Micronization of Glucagon 

Using a mortar and pestle, the glucagon particles were reduced to inhalable size (0.5–2 µm) and confirmed by scanning electron microscopy (SEM). 

#### 2.2.2. Preparation of Interactive Mixtures and Homogeneity Test

Interactive powder mixtures of 2.5% glucagon, 5% leucine, MgSt, and lactose (Inhalac 120, volume median diameter 157 µm) were prepared by a validated hand mixing method [24,25]. Briefly, the powder mixtures of glucagon (2.5%) and lactose were prepared in 2.5 g batches (62.5 mg glucagon and 2.44 g of lactose). The drug particles were placed between two layers of lactose carrier in a glass test tube with three glass beads (10 mm in diameter). After ensuring proper stoppering, the test tube was vigorously shaken by hand for 5 min. Similarly, ternary mixtures of this drug (2.5%) with lactose and other excipients were prepared by separately mixing leucine (5%) and MgSt (5%). The glass beads provided a ball-milling effect to break up any agglomerates formed during powder mixing. The homogeneity of each powder mixture was assessed by the mean drug content and variability between samples. Five randomly selected samples of 10 mg each were dissolved in 0.016% HCl solution. An acceptable degree of homogeneity was achieved with a mean drug content with a coefficient of variation of less than 4% for all samples [26].

#### 2.2.3. Drug Dispersion by Twin-Stage Impinger (TSI) 

The powder formulations were filled (~20 mg) into the hard gelatin capsules (size 3, Fawns and McAllan Pty Ltd., Belmont, Australia) manually. The in-vitro aerosol deposition of the powder formulations delivered from a Rotahaler^®^ (Allen and Hanburys, Middlesex, UK) was determined using a twin-stage impinger (TSI, Apparatus, A; British Pharmacopoea, 2000) (Copley, UK). Quantities of 7 and 30 mL of 0.016% HCl solution were placed into stage one (S1) and stage two (S2) of the TSI, respectively. The air flow was drawn through the TSI using a vacuum pump, and the air flow rate was adjusted to 60 ± 5 L/min at the mouthpiece prior to each measurement. After each experiment, the Rotahaler (including the capsule shells) and S1 and S2 compartments were washed separately with the solvent, and the deposition of drug particles in different stages of TSI was measured using LC-MS/MS. The glucagon particle depositions of different formulations were evaluated based on the three parameters: the recovered dose (RD), the emitted dose (ED), and the fine particle fraction (FPF). The FPF was defined as the percentage of the RD deposited in the lower stage (S2) of the TSI. The RD was the total amount of drug collected from the Rotahaler (device, mouthpiece, and capsule) and the S1 and S2 compartments. The ED was the percentage of RD delivered from the Rotahaler. 

#### 2.2.4. Quantitative Analysis of Glucagon by LC-MS/MS

Liquid chromatographic (LC) separation and mass spectrometric (MS) detection were performed using the Sciex Triple Quad QTRAP^®^ 4500 LC-MS/MS system coupled with a Shimadzu LC System (AB Sciex, Singapore). The samples were collected and then either centrifuged (5 min at 9000× *g*) or filtered. The supernatants or filtrates were assayed for glucagon analysis. A calibration curve was prepared in water (with 0.016% HCl, pH = 3) and spiked with glucagon to give seven calibration standards ranging from 1.25 to 200 µg /mL and 3 levels of control (2.5, 25, and 150 µg /mL). HPLC separation was achieved using a Phenomenex C18 analytical column (100 × 2.1 mm, i.d.; 1.7 µm). The mobile phase, flowing at a rate of 0.3 mL/min, was made of 0.1% formic acid in water, and acetonitrile with water mixed in a volume ratio of 80:20. The total run time was 5 min. Five microliter samples were directly injected without dilution. The MS was operated using positive electrospray ionization with Analyst 1.6 software (AB Sciex, Singapore). Quantitation was performed by precursor-product ion transitions in multiple reaction monitoring (MRM) mode at *m/z* 871.3 → 217.2 with a dwell time of 20 ms per transition. The limit of detection (LOD) and the limit of quantitation (LOQ) were 50 ng/mL and 1.25 µg/mL, respectively. Linear coefficient values were >0.999, as calculated using MultiQuant 3.0. (AB Sciex, Singapore) Intra- and inter-day variabilities were characterized at three levels of concentration, with the precision and accuracy always being lower than 15%. For the calculation of the slope and intercept of the calibration curve, a non-weighted linear regression was applied.

#### 2.2.5. Morphological Studies by SEM 

The morphological properties of all samples were examined by SEM (Jeol JSM-6360A, Tokyo, Japan). A small amount of dried powder was sprinkled onto a silicon wafer adhered to an aluminum stub through double-sided carbon adhesive tape. The air-dried specimen stubs were coated with a conductive layer of sputtered gold (Leica, argon gas pressure of 0.5 mbar, current of 30 mA, and a coating time of 75 s), followed by observing secondary electron images under a high vacuum with an accelerating voltage of 5 kV and a working distance of 6.8 mm. 

#### 2.2.6. ATR-FTIR 

The ATR-FTIR spectra of glucagon in all formulations were obtained using a Thermo Nicolet Nexus 870 FTIR spectrometer (Triad Scientific Inc., Manasquan, NJ, USA) equipped with a single reflection diamond crystal attenuated total reflectance (ATR) accessory with an angle of incidence of 40° and a deuterated triglycine sulfate (DTGS). A small amount of sample was placed on the top of the diamond crystal and secured with a high-pressure clamp. The spectra were collected at a resolution of 8 cm^−1^, and 64 scans were performed in the range of 4000–500 cm^−1^ and analyzed using the spectral analysis software OMNIC (Nicolet Instrument Corp., Version 7.2, Madison, WI, USA). To understand the chemical integrity of glucagon in all formulations, the characteristic bands relating to the amide I region (α-helix), which is located between 1700 and 1600 cm^−1^ has often been used in literature, as it is based on the vibration of only few molecules [27]. Therefore, in this study, the FTIR band spectra of amide I was examined to investigate the structural integrity of the glucagon in different formulations. To obtain the FTIR spectra of glucagon in the powder mixtures, appropriate subtraction of the excipients’ (lactose, MgSt and leucine) spectra were carried out in order to generate a straight baseline in the amide I region.

#### 2.2.7. Differential Scanning Calorimetry (DSC)

Using a DSC Q100 TA Q series (TA Instruments Inc., New Castle, DE, USA), the thermal properties of all formulations were determined. A small amount of sample (<5 mg) was enclosed in a hermetic sealed aluminum pan, and all formulations were scanned from 20 to 250 °C at a heating rate of 10 °C/min. The stability of glucagon in different formulations was determined using the characteristic prominent DSC peak at 70 °C, which represents the melting point of glucagon.

#### 2.2.8. Raman Spectroscopy 

A Raman microscope (WITec Alpha 300 series, Ulm, Germany) equipped with a 532 nm laser was used for spectral analysis of the individual components and the mixture of glucagon with other excipients. The Raman maps were measured by rastering in the horizontal plane in 0.5 μm increments over a 50 × 50 μm area of the powder mixture with the focal plane of the microscope set to just beneath the coverslip. Emphasis was given to the identification of a large lactose crystal on which other excipients were adhered. Spectra were recorded at each increment with an integration time of 1 s and a laser power of 10 mW. The Zeiss 50× objective using 0.7 NA formed a confocal sample volume that approximated a cylinder of 0.5 μm diameter and 2–3 μm height. The mapping of the powder in the mixture was performed using WITec Control Four and Project Four software (WITec GmbH, Ulm, Germany).

#### 2.2.9. Statistical Analysis

All statistical analyses were performed using one-way ANOVAs in Microsoft Excel (2016).

## 3. Results and Discussions

### 3.1. FTIR Analysis

**Figure 1:**
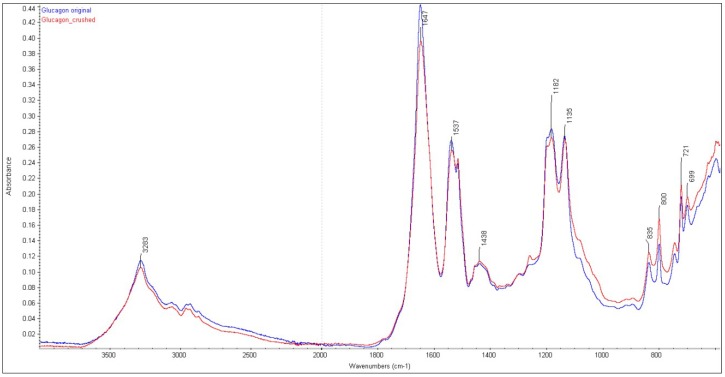
ATR-FTIR spectra of glucagon and their mixtures.

The FTIR of the original and processed (ground for making inhalable size, <5 µm) glucagon hydrochloride showed the characteristic amide I band at 1649 cm^−1^ and 1647 cm^−1^ (Figure 1). The regions of these bands were slightly different from spectra previously recorded by others [21,22]. Using IR, Fang et al. found a characteristic peak at 1657 cm^−1^ associated with the amide I band in freeze-dried glucagon containing glycine hydrochloride buffer. Stigsnaes et al. found a characteristic FTIR amide I band of glucagon at 1655 cm^−1^ [22], whereas Vonhoff et al [23] found this band at 1656 cm^−1^. The reason behind this variation is not clear; however, it is probable that the conformation of the protein in the hydrochloric acid salt form might have affected the physical properties. Using FTIR, the PEGylated freeze-dried glucagon showed a significantly improved physical stability, possibly due to steric hindrance of peptide–peptide interactions [22].

**Figure 2:**
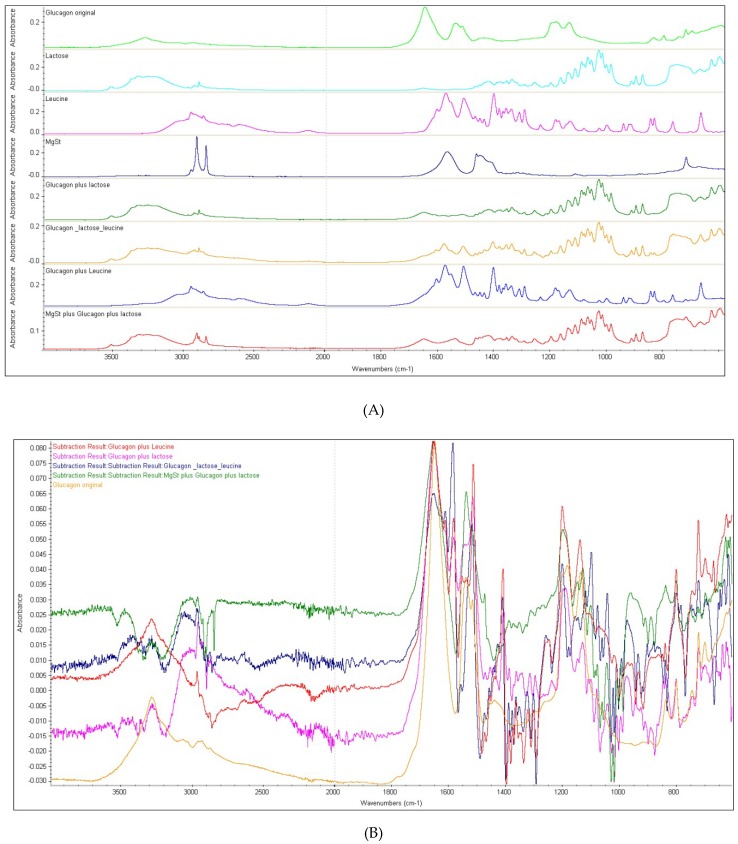
(**A**) ATR-FTIR spectra of all raw materials and powder mixtures; (**B**) ATR-FTIR spectra of the original glucagon and the subtracted glucagon from the mixture with leucine.

The FTIR spectra of the mixtures of glucagon with excipients (lactose, leucine, and MgSt) are presented in Figure 2A, and different spectra patterns can be observed. To understand the interactions between glucagon and lactose, leucine, and their mixtures, subtraction of glucagon spectra from the spectra of powder mixtures was performed and the resulting spectra were compared with the glucagon spectra (Figure 2B). It was apparent that there were some interactions between glucagon and leucine, as evidenced by the presence of extra peaks between 3000 to 2800 cm^−1^ (Figure 2B). These peaks could be due to the changes in the positions of OH groups, i.e., hydrogen bonding between glucagon and leucine. The appearance of a new peak at 1585 cm^−1^ also demonstrated their interactions in the mixture. With regard to the interaction of glucagon with large carrier lactose, the subtracted glucagon showed major changes between 3035 and 2868 cm^−1^ (Figure 2B). This suggests that the glucagon somehow associated with lactose, possibly by forming hydrogen bonds. It should be noted here that the structural integrity of glucagon in the mixture of both leucine and lactose was remarkably affected (Figure 2B, glucagon subtracted from the mixture) and this can be seen by the presence of broad peaks between 3487 and 2890 cm^−1^, which indicate that the glucagon interacted with both lactose and leucine due to the formation of hydrogen bonding between them. Therefore, this result suggests that the leucine is not a suitable excipient to be used in glucagon DPI formulation with a lactose carrier.

**Figure 3:**
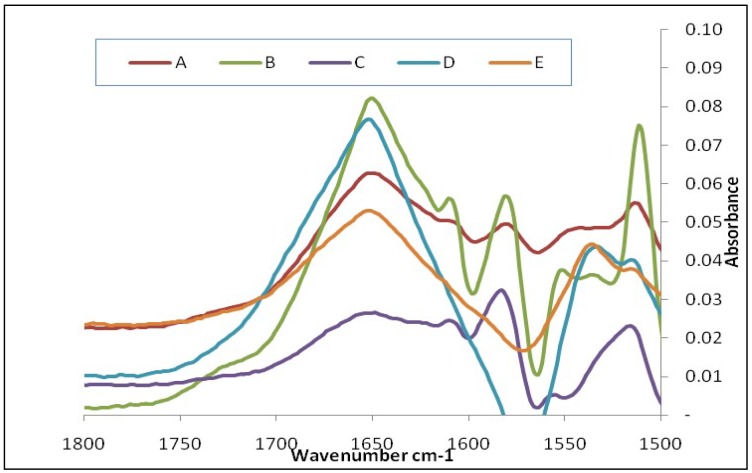
The subtracted FTIR spectra of glucagon hydrochloride from different mixtures (enlarged view in the amide I region). Glucagon subtracted from the mixtures of lactose (**A**); leucine (**B**), lactose and leucine(**C**), magnesium stearate (**D**), and lactose and magnesium stearate (**E**).

To further understand the changes in the amide I region, all glucagon-subtracted spectra and the pure glucagon spectrum are presented in Figure 3. Glucagon, in combination with either lactose or leucine, showed the characteristic amide I peak (peak at 1652 cm^−1^). The drug mixed with large carrier lactose showed an additional weak shoulder peak at 1612 cm^−1^ (Figure 3A), which could be evidence of a glucagon–lactose interaction in the formulation. While considering the interaction between glucagon and leucine, the appearance of a sharp peak at 1612 cm^−1^ and a very weak shoulder at 1625 cm^−1^ (Figure 3B) suggest that the leucine had a significant impact on the stability of glucagon in the mixture. The combination of lactose and leucine with glucagon had a significant impact on the structural integrity of glucagon, which produced a very broad peak (weak intensity) at 1651 cm^−1^ and new peaks at 1612 cm^−1^ (sharp), 1625 cm^−1^, and at 1638 cm^−1^ (Figure 3C), which indicated a significant interaction of glucagon with leucine and lactose, as demonstrated earlier. This outcome suggests that the dispersibility enhancer leucine was not compatible with glucagon in the DPI formulation. With regard to MgSt, glucagon with either MgSt or in the combination of MgSt and lactose showed a characteristic amide peak at 1654 cm^−1^; however, no additional peaks were observed around the amide I region (Figure 3D,E). This indicates that there were no interactions between these excipients and glucagon in the formulation containing MgSt and lactose which can be considered the most stable formulation. As lactose in the presence of MgSt did not show any signs of interactions, it can be concluded that leucine interacted with glucagon in the formulation rather than with lactose. Although freeze-dried glucagon with excipients, such as trehalose, hydroxyethyl ester, polyethylstyrene, and polysorbate 20, has shown no remarkable changes in the band spectra of amide I by others, trehalose and polysorbate induced chemical and physical stability in glucagon [21].

### 3.2. Crystallinity of Powders 

**Figure 4:**
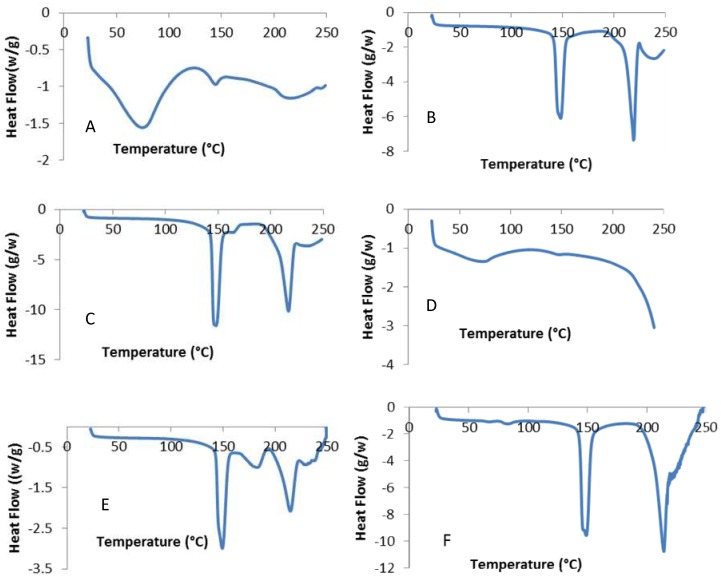
DSC spectra of (**A**) glucagon hydrochloride, (**B**) lactose monohydrate, (**C**) mixture of glucagon and lactose monohydrate, (**D**) mixture of glucagon and leucine, (**E**) mixture of glucagon, leucine, and lactose monohydrate; (**F**) mixture of glucagon, magnesium stearate, and lactose monohydrate.

The DSC data of all formulations are presented in Figure 4. The active ingredient glucagon hydrochloride showed a prominent characteristic endothermic peak at 70.8 °C, a second weak peak at 143.7 °C, and a third broad peak 219.5 °C. Glucagon was shown to be highly unstable, and the HCl salts of most of the compounds were less stable due to their amorphous nature. Although it is not clearly known, the first two peaks are related to the loss of water, and the third peak is associated with the melting of glucagon. We suggest that the observed enthalpy change with increased heat flow was due to the amorphous content in the drug particles (Figure 4A). All of these peaks of glucagon disappeared in the formulation containing large carrier lactose with and without leucine (Figure 4B,C), and with leucine only (Figure 4D), which is an indication of interaction between the glucagon and leucine. The DSC thermograms of lactose carrier indicated the presence of α-lactose monohydrate, as demonstrated by the presence of endothermic peaks in the range of 141 to 147 °C that corresponded to the release of water from crystallization as well as an endothermic decomposition peak in the range of 207 to 219.9 °C (Figure 4B) which represented the melting of α-lactose monohydrate [28,29]. These findings suggest that there was no change in the lactose particles in the presence of leucine and glucagon in the formulation. The mixture of leucine with glucagon showed a very weak and broad peak at 75 °C (Figure 4D); however, no peaks of glucagon were observed in the mixture with lactose alone (Figure 4C) or with the combination of both leucine and lactose (Figure 4E). This clearly indicates that the crystallinity change of glucagon occurred in the presence of leucine or lactose, thus confirming their interaction with glucagon in the formulations. The reason for the glucagon interaction with leucine was not clear; however, as previously demonstrated, physical interactions through the formation of hydrogen bonds, as confirmed by FTIR, might have affected the stability of glucagon in the mixture. However, the FTIR and Raman data showed no evidence of chemical degradation of glucagon in those mixtures. The formulation containing glucagon, MgSt, and lactose, showed a very weak characteristic peak of glucagon at 65.2 °C and another broad peak at 81.9 °C (Figure 4F). These peaks were within the range of the glucagon peak, which demonstrates that there was no interaction between the glucagon and MgSt or lactose. The FTIR and DSC data confirmed that MgSt with lactose was compatible with glucagon and could be considered suitable carriers for the glucagon DPI formulation. The observed weak peak was due to the low concentration of glucagon (only 2.5%) in the formulation. The peak for leucine alone, although not required, was unable to be detected in the temperature range of 20–250 °C. 

### 3.3. Raman Mapping

**Figure 5:**
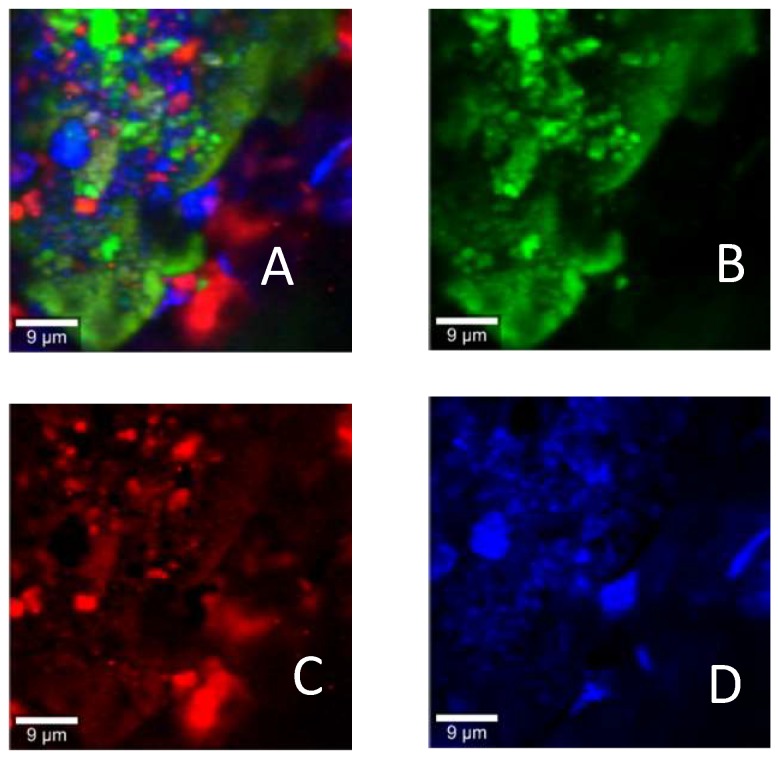
Raman images of (**A**) powder mixture, (**B**) lactose, (**C**) glucagon, and (**D**) leucine. All excipients (glucagon and leucine) adhered to the surface of large carrier lactose.

**Figure 6:**
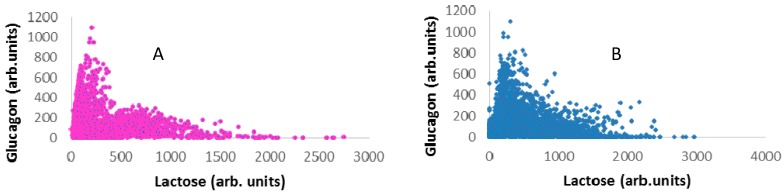
Raman correlation plots of the mixture of glucagon with the lactose carrier (**A**) and glucagon with leucine (**B**).

Using Raman spectroscopy, the distribution and identification of individual components in the powder mixtures was determined, as presented in Figure 5, where image A was formed by merging the component images to give an overall impression of distribution of all components in the powder mixture. The Raman images revealed that glucagon drug particles were adhered to the surface of lactose; however, they were surrounded by leucine (Figure 5A). The distribution of glucagon on the large carriers was related to the association between L-leucine and glucagon. To understand the degree of possible associations between lactose and leucine with glucagon, correlation diagrams were prepared by plotting the relative concentration of each component vs. the glucagon concentration for each mapped point (Figure 6A,B). Both figures showed very high positive correlations between glucagon and lactose or leucine, which were very closely associated with the drug particles. The hydrophilic nature of both lactose and leucine (amphiphilic) led to affinity with glucagon owing to the presence of a hydroxyl group to form hydrogen bonds. These outcomes provide a clear understanding of the excipients’ interactions and associations with glucagon, which could be used to develop better glucagon DPI formulations. 

## 4. Aerosolization of Glucagon from DPI Formulations

**Figure 7:**
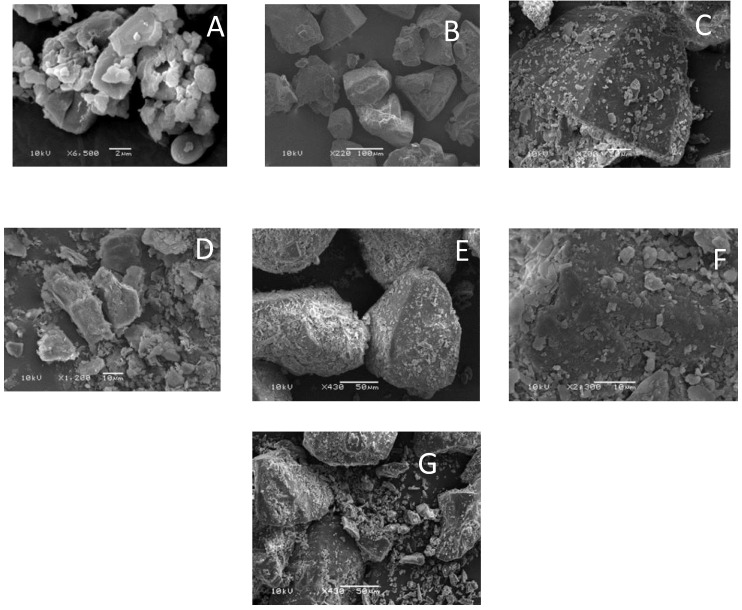
**(A**) Micronized glucagon; (**B**) lactose; (**C**) mixture of lactose and glucagon; (**D**) leucine; (**E**) mixture of lactose, glucagon and leucine; (**F**) close up view of E; (**G**) mixture of lactose, glucagon, and MgSt.

The dispersion behaviors of glucagon hydrochloride from different formulations are presented in Table 1. All formulations showed that the ED values were 48–62%, which indicates that particles were not effectively emitted from the devices. The FPF of the drug only formulation was 6.4%, which is extremely low compared to those of available DPI products. The poor FPF of the drug could be due to the cohesive nature of the micronized drug particles (0.5–2 µm). To overcome this poor flow behavior, the micronized glucagon powder was mixed with a large carrier (Inhalac 120, size around 90 µm) to enhance the flow properties of powder. This subsequently increased the FPF to 32%, which was more than 5-fold higher than that of the carrier-free formulation. It is well-known that large carrier-based formulations produce higher FPF values, as large carriers reduce the cohesive forces among inhalable particles by improving de-agglomeration due to the collision effects [30,31]. Because of the higher mass of large carriers, the impaction of particles with the device wall was very high, which caused greater separation of drug particles from the surfaces of large carriers. To increase the FPF further, we used a well-known dispersibility enhancer, leucine, with the large carrier in the formulation, which produced a higher FPF (35.5%) of glucagon compared to the formulation without leucine (Table 1). Although it was expected that the formulation with leucine would give a higher FPF, practically, leucine showed a poor performance in dispersing the drug from the formulation. The reason for this was the physical interaction of glucagon with leucine, as confirmed by the FTIR and DSC studies. Both the glucagon and leucine have amino groups as well as hydroxyl groups, which might interact with each other by hydrogen bonding. The FTIR data showed a physical interaction between glucagon (the band spectra for amide I shifted from 1647 to 1652 cm^−1^, Figure 3) and leucine, which might have changed the integrity of the glucagon structure in the mixture. The DSC data also supported the interactions between glucagon and leucine, as there was no peak associated with glucagon at 70.8 °C. Moreover, around 50% of drugs were retained in the device (i.e., 50% was dispersed) during dispersion analysis, even though the pressure was maintained at 60 L/min. Furthermore, the standard deviations of both ED and FPF were high, which also proved that the powder flow property changed in the presence of leucine in the formulation. Therefore, it is likely that these physical interactions/association reduced the powder flow, which affected the FPF of glucagon. The SEM image (Figure 7) of the formulation showed that leucine formed complex agglomerates with glucagon particles that were off the surface of large carriers, leading to reduced dispersion of drug particles compared to the formulation without leucine. This finding confirmed that the leucine was not compatible with glucagon and therefore not suitable for glucagon DPI formulation. To understand more about the excipient interactions, MgSt was used in the formulation, and a similar FPF (36%) was obtained; however, the FPF was significantly higher compared to that of the formulation without excipients. Although the FPFs of formulations containing MgSt and leucine produced similar FPF values, it should be highlighted here that the drug powder with MgSt showed 66% ED, which was significantly higher than that of powder with leucine. Furthermore, the dispersibility behavior was consistent, as evidenced by the low standard deviations. The FTIR, as well as the DSC data, showed no interactions with glucagon, as the FTIR band spectra of amide I appeared at 1654 cm^−1^ and the characteristic melting point peak for glucagon was also observed around 70 °C. The FTIR spectra of the low frequency β-sheet found to be shifted (1638 cm^−1^-1612 cm^−1^) in the mixtures of glucagon with lactose and leucine (Table 2) due to physical interactions; however, no such remarkable changes occurred in amide I band. Therefore, it could be emphasized that the low and variable FPF value of glucagon from the mixture of glucagon, leucine, and lactose was due to the physical interactions, which reduced the dispersibility of the drug. In contrast, the MgSt improved the FPF without any interactions with glucagon (FTIR spectra showed no changes in the low frequency β-sheet, Table 2), and thus, the results conclude that the dispersibility enhancer MgSt along with the carrier lactose is compatible with the glucagon in DPI formulation. 

## 5. Conclusions

Using some universal excipients, such as lactose as a carrier, and leucine and MgSt as dispersibility enhancers, several DPI formulations of glucagon for pulmonary delivery were developed and characterized. FTIR, DSC, and Raman confocal microscopy technologies were successfully employed to characterize the formulations and the interactions of glucagon with excipients in the mixtures. This study is the first to report that leucine could have a significant physical interaction with glucagon, as confirmed by FTIR, DSC, and Raman spectroscopies. The FTIR, Raman, and DSC data also showed no interactions between glucagon and MgSt, and the mixture of glucagon, MgSt, and lactose. The highest FPF (36%) of glucagon was obtained from the formulation containing MgSt and lactose. L-leucine showed a physical interaction and resulted in a reduction in the FPF of glucagon. Therefore, the results reported here confirm that leucine is not a suitable dispersibility enhancer for glucagon DPI formulations; however, the mixture of glucagon with lactose and MgSt was found to be an efficient glucagon DPI formulation with significantly improved dispersion. The FTIR, DSC, and Raman confocal microscopy techniques were found to be very useful tools for characterizing the drug–excipient interactions and could be used to select suitable excipients for the development of a stable glucagon DPI formulation.

## Figures and Tables

**Table 1:** Glucagon dispersion from dry powder inhaler (DPI) formulations. RD: recovered dose; ED: emitted dose; FPF: fine particle fraction.

Samples	RD (%)	ED (%)	FPF (%)
Glucagon (2.5%) plus lactose	95.9 (7.1)	62.7 (2.9)	31.7 (2.7)
Glucagon (2.5%) plus 5% leucine plus lactose	100.9 (2)	52.6 (8)	35.5 (4.9)
Glucagon (2.5%) plus 5% magnesium stearate plus lactose	101.9 (3.7)	66.3 (3.5)	36.3 (1.3)
Glucagon only	102.8 (0.7)	48.2 (10.9)	6.4 (0.8)

NB: Standard deviations from triplicates were within the brackets.

**Table 2:** Band in the amide I region of FTIR spectra of glucagon in different formulations.

Samples	Low Frequency β-Sheet	α-Helix
Glucagon original	-	1649 cm^−1^
Glucagon inhalable particles	-	1647 cm^−1^
Glucagon subtracted from leucine	1612 cm^−1^ 1625 cm^−1^	1651 cm^−1^
Glucagon subtracted from lactose		1652 cm^−1^
Glucagon subtracted from the mixture of lactose plus leucine plus glucagon	1612 cm^−1^ 1624 cm^−1^ 1638 cm^−1^	1651 cm^−1^
Glucagon subtracted from MgSt	-	1654 cm^−1^
Glucagon subtracted from the mixture of lactose plus MgSt plus glucagon	-	1654 cm^−1^
